# Immunoprotection evaluation of the recombinant N-terminal domain of Cys-loop receptors against *Rhipicephalus (Boophilus) microplus* tick infestation

**DOI:** 10.1051/parasite/2021064

**Published:** 2021-09-14

**Authors:** Moisés Martínez Velázquez, Carla Patricia Barragán Álvarez, José Miguel Flores Fernández, Rodolfo Esteban Lagunes Quintanilla, Edgar Castro Saines, Patricia Berenice Ramírez Rodríguez, Sara Elisa Herrera Rodríguez, Rodolfo Hernández Gutiérrez, Abel Gutiérrez Ortega, Ángel H. Álvarez

**Affiliations:** 1 Centro de Investigación y Asistencia en Tecnología y Diseño del Estado de Jalisco, AC Av. Normalistas 800, Col. Colinas de la Normal 44270 Guadalajara Jalisco México; 2 Centro Nacional de Investigaciones en Parasitología Veterinaria-INIFAP Carretera Federal Cuernavaca Cuautla 8534, Col. Progreso 62550 Jiutepec Morelos México

**Keywords:** *Rhipicephalus microplus*, Cys-loop receptor, Tick, Vaccine, Cattle

## Abstract

*Rhipicephalus (Boophilus) microplus* ticks are obligatory hematophagous ectoparasites of cattle and act as vectors for disease-causing microorganisms. Conventional tick control is based on the application of chemical acaricides; however, their uncontrolled use has increased resistant tick populations, as well as food and environmental contamination. Alternative immunological tick control has shown to be partially effective. Therefore, there is a need to characterize novel antigens in order to improve immunological protection. The aim of this work was to evaluate Cys-loop receptors as vaccine candidates. N-terminal domains of a glutamate receptor and of a glycine-like receptor were recombinantly produced in *Escherichia coli*. Groups of BALB/c mice were independently immunized with four doses of each recombinant protein emulsified with Freund’s adjuvant. Both vaccine candidates were immunogenic in mice as demonstrated by western blot analysis. Next, recombinant proteins were independently formulated with the adjuvant Montanide ISA 50 V2 and evaluated in cattle infested with *Rhipicephalus microplus* tick larvae. Groups of three European crossbred calves were immunized with three doses of each adjuvanted protein. ELISA test was used to evaluate the IgG immune response elicited against the recombinant proteins. Results showed that vaccine candidates generated a moderate humoral response on vaccinated cattle. Vaccination significantly affected the number of engorged adult female ticks, having no significant effects on tick weight, egg weight and egg fertility values. Vaccine efficacies of 33% and 25% were calculated for the glutamate receptor and the glycine-like receptor, respectively.

## Abstract (fr)

Les tiques *Rhipicephalus (Boophilus) microplus* sont des ectoparasites hématophages obligatoires des bovins et agissent comme vecteurs de micro-organismes pathogènes. Le contrôle conventionnel des tiques est basé sur l’application d’acaricides chimiques, mais leur utilisation incontrôlée a augmenté les populations de tiques résistantes ainsi que la contamination des aliments et de l’environnement. Le contrôle immunologique alternatif des tiques s’est avéré partiellement efficace. Par conséquent, il est nécessaire de caractériser de nouveaux antigènes afin d’améliorer la protection immunologique. Le but de ce travail était d’évaluer les récepteurs Cys-loop comme candidats vaccins. Les domaines N-terminaux d’un récepteur du glutamate et d’un récepteur de type glycine ont été produits par recombinaison chez *Escherichia coli*. Des groupes de souris BALB/c ont été immunisés indépendamment avec quatre doses de chaque protéine recombinante émulsionnée avec l’adjuvant de Freund. Les deux vaccins candidats étaient immunogènes chez la souris, comme l’a démontré l’analyse par transfert Western. Ensuite, des protéines recombinantes ont été formulées indépendamment avec l’adjuvant Montanide ISA 50 V2 et évaluées chez des bovins infestés de larves de tiques *Rhipicephalus microplus*. Des groupes de trois veaux croisés européens ont été immunisés avec trois doses de chaque protéine avec adjuvant. Le test ELISA a été utilisé pour évaluer la réponse immunitaire IgG induite contre les protéines recombinantes. Les résultats ont montré que les candidats vaccins généraient une réponse humorale modérée sur les bovins vaccinés. La vaccination a affecté de manière significative le nombre de tiques femelles adultes engorgées mais n'a eu aucun effet significatif sur le poids des tiques, le poids des œufs et les valeurs de fertilité des œufs. Des efficacités vaccinales de 33 % et 25 % ont été calculées pour le récepteur du glutamate et le récepteur de type glycine, respectivement.

## Introduction

*Rhipicephalus (Boophilus) microplus* tick infestations deeply affect cattle production because these parasites cause damage directly in addition to acting as vectors for disease-causing agents such as *Anaplasma* spp. and *Babesia* spp. [33]. Chemical control remains the tick control method of choice [27]. However, this practice regularly leads to the selection of tick populations resistant to the chemical used to treat infested cattle [16, 29, 32]. Immunological control has emerged as a promising alternative to restrain tick infestations and pathogen transmission.

This new approach prompted the development and commercialization of vaccines based on the recombinant protein Bm86 [7, 21, 31, 36]. This success was, however, overshadowed by the fact that these vaccines show variable effectiveness in different tick populations, attributable to antigenic variations in the Bm86 protein between tick populations [7, 19, 37]. Consequently, there is a need to characterize novel antigens in order to improve immunological protection.

The reverse vaccinology approach is a promising strategy for the identification of novel anti-tick vaccine candidates. Candidates are selected based on their expression level, subcellular localization, accessibility and biological function, among other factors [26]. Hence, we focused our attention on members of the Cys-loop ligand-gated ion channels (LGICs) family from *R. microplus* tick. Cys-loop receptors are membrane-spanning proteins and they play key biological functions mediating the synaptic transmission of nerve impulses [22]. We have previously shown that genes encoding for a glutamate-gated receptor and a glycine-like receptor are expressed at egg, larval and adult developmental stages of *R. microplus* [17, 18]. Both receptors are membrane-spanning neurotransmitter-gated ion channels that are responsible for fast transmission in the peripheral and central nervous systems. They share a common structure of five subunits. Each subunit has a large N-terminal extracellular domain (ECD), four transmembrane domains (TMD), a large intracellular domain (ICD) and a short C-terminal ECD [41]. Being proteins located in the cell membrane, their extracellular antigenic regions would be easily accessible to antibodies produced by an immunized bovine host.

In the present study, we followed the reverse vaccinology approach and applied the above-mentioned selection criteria to decide on and assess the immunogenicity and protective efficacy of recombinant N-terminal ECD of a glutamate-gated receptor [20] and of recombinant N-terminal ECD of a glycine-like receptor [17] from *R. microplus* tick.

## Materials and methods

### Production of recombinant proteins

Nucleotide sequences encoding for the N-terminal ECD of a glutamate-gated receptor (GluCl; GenBank accession no. KF881800) and the N-terminal ECD of a glycine-like receptor (GlyR; GenBank accession no. KJ476181) from *R. microplus*, were commercially synthesized by Genscript (Piscataway, NJ, USA) and subcloned from pUC57 plasmid into *Nco*I/*Xho*I sites of pET32b(+) plasmid (Merck Millipore, Burlington, MA, USA). These constructs were used to transform electro-competent *Escherichia coli* BL21 (DE3) cells (Thermo Fisher Scientific, Waltham, MA, USA). Transformants were selected by antibiotic resistance marker and plasmid insertion was confirmed by restriction enzyme digestions. Transformed colonies were grown in 1 L flasks containing Luria-Bertani broth to an optical density (OD) at 600 nm of 0.8–1, at 37 °C and 250 rpm. Protein expression was induced with 1 mM isopropyl-β-d-thiogalactopyranoside (IPTG) for 4 h. Then, bacteria were harvested, lysed by sonication using an ultrasonic homogenizer Microson XL-2000 (Misonix, Inc., Farmingdale, NY, USA) in phosphate-buffered saline (PBS) with 0.1% Triton X-100, and cell debris separated by centrifugation. Recombinant proteins were His-tag metal affinity purified using 5 mL Bio-Scale Mini Profinity IMAC cartridges (Bio-Rad Laboratories, Philadelphia, PA, USA), which had been previously equilibrated with buffer A (300 mM KCl, 50 mM KH_2_PO_4_, 5 mM imidazole). After two consecutive washes with one column volume of buffer A, bound proteins were eluted in buffer A supplemented with 250 mM imidazole. Then, eluted proteins were subjected to SDS-PAGE in 12% polyacrylamide gels, visualized by the zinc/imidazole reverse staining procedure, excised from the gels, and finally recovered from polyacrylamide matrix by electroelution using an Electro-Eluter (Bio-Rad Laboratories, Philadelphia, PA, USA). Recombinant proteins in elution buffer were mixed with 1% Triton X-114, chilled on ice and warmed at 37 °C until formation of two phases. After centrifugation, upper aqueous phase with endotoxin free proteins was recovered and residual detergent was removed by dialysis with 25 mM HEPES-10% glycerol-150 mM NaCl. Specific molecular weight of recombinant proteins was confirmed by Western blot after SDS-PAGE in 10% polyacrylamide gels using a mouse anti-His tag monoclonal antibody (Roche Molecular Biochemicals, Indianapolis, IN, USA).

### Mice immunization

Seven 6-week-old female BALB/c mice were obtained from Harlan Laboratories (Mexico City, Mexico) and maintained in cages with food and water *ad libitum*. They were divided into 2 groups of 4 and 3 mice, respectively. Groups were immunized with 20 μg of recombinant N-terminal ECD of glutamate-gated receptor (rGluCl, 4 mice) or recombinant N-terminal ECD of glycine-like receptor (rGlyR, 3 mice) mixed with complete Freund’s adjuvant (Sigma-Aldrich, St. Louis, MO, USA) for priming and incomplete Freund’s adjuvant (Sigma-Aldrich, St. Louis, MO, USA) for boosting. Immunizations were carried out on days 1, 15, 30 and 45. Blood was collected by tail vein bleeding before immunization (bleeding 1), 5, 42 and 50 days after first immunization (bleedings 2, 3 and 4, respectively). Serum samples were obtained and preserved at −80 °C until use. Mice care and maintenance were carried out in the Vaccine Evaluation Module of Animal Experimentation Facilities (Centro de Investigación y Asistencia en Tecnología y Diseño del Estado de Jalisco, México), following international protocols.

### Western blot assays

Western blot assays were carried out to determine the immunogenicity of rGluCl and rGlyR proteins in mice. Briefly, recombinant proteins were separately electrophoresed on 12% polyacrylamide preparative gels and transferred to nitrocellulose membranes. Membranes were blocked in PBS solution containing 0.05% Tween 20 (PBS-T) and 5% skim milk for 2 h at 4 °C, with gentle rocking. After that, membranes were washed three times in PBS-T, cut into strips (~0.4 mm) and incubated with different mice sera diluted 1:100 in PBS-T-milk in mini incubation trays (Bio-Rad Laboratories, Philadelphia, PA, USA) for 24 h at 4 °C. Later, strips were washed three times with PBS-T and incubated with HRP-conjugated goat anti-mouse IgG (Bio-Rad Laboratories, Philadelphia, PA, USA) as secondary antibody, diluted 1:3000 in PBS-T-milk for 4 h, at room temperature, followed by washing with PBS-T solution. Finally, immunoreactive bands were evidenced using an HRP Substrate and Detection kit (Opti-4CN; Bio-Rad Laboratories, Philadelphia, PA, USA).

### *Rhipicephalus microplus* tick strain

The multiresistant *R. microplus* tick strain (Isla strain, Mexico) was obtained from a laboratory colony maintained at CENAPA-SENASICA, Jiutepec, Morelos, Mexico. Originally, these ticks were collected from infested cattle in Isla ranch, Padilla municipality, Tamaulipas, Mexico, and reported to be resistant to chlorfenvinphos, coumaphos, diazinon, lindane, cypermethrin, deltamethrin and flumethrin [6]. Tick larvae were fed on cattle and collected after repletion and kept under controlled laboratory conditions for oviposition and hatching in humidity chambers at 12 h light: 12 h dark photoperiod, 25–27 °C and 80% relative humidity (RH). Larvae were ~20 days of age at the time of infestations.

### Cattle immunization and tick infestations

The experimental protocol was approved by the Institutional Committee of Animal Experimentation of the National Animal Health Verification Services Center (CENAPA, México; approval number: PE01/16). Cattle were individually housed in isolation pens and were fed on fodder receiving water *ad libitum*. Three European crossbred calves per group were each immunized with 3 doses (days 1, 30 and 50) containing 100 μg/dose of rGluCl or rGlyR proteins. A negative control group (three animals) was injected with adjuvant/saline alone. Emulsions were made with recombinant proteins in PBS plus adjuvant Montanide ISA 50 V2 (anhydromannitoletheroctodecenoate; Seppic, Paris, France) at 1:1 volume ratio. Cattle were injected intramuscularly with 2 mL/dose using a 5 mL syringe and an 18G needle. Two weeks after the second immunization, cattle in the vaccinated and control groups were infested with ~1850 *R. microplus* (Isla strain, Mexico) larvae/animal, and thereafter every third day, totalizing 18 infestations. Cattle were cared for in accordance with standards specified in the Guide for Care and Use of Laboratory Animals.

### Cattle blood collection

Blood samples were collected from cattle every week for 12 weeks. Those obtained on weeks 1, 5 and 8 were collected before immunization. Samples were collected from the caudal vein into sterile tubes using vacutainer needles (0.8 × 38 mm; BD, Franklin Lakes, NJ, USA) and maintained at 4 °C until arrival at the laboratory. Serum was separated from cellular components by centrifugation at 5000 rpm for 1 min and stored at −20 °C until used. Antibody levels were determined using an antigen-specific indirect enzyme-linked immunosorbent assay (ELISA).

### Determination of serum antibody levels by ELISA

Purified rGluCl and rGlyR proteins were diluted in a coating buffer (0.05 M carbonate bicarbonate buffer, pH 9.6) and used to coat 96-well ELISA plates (0.1 μg/well; Nunc MaxiSorp, Thermo Fisher Scientific, Waltham, MA, USA), which were then incubated overnight at 4 °C. The plates were washed three times with wash buffer (PBS-T) and then blocked with a blocking buffer (5% skim milk in PBS). Plates were incubated with cattle serum samples diluted 1:100 in PBS-T for 1 h at 37 °C. After washing three times with washing buffer, plates were incubated with HRP-conjugated goat anti-bovine IgG (Sigma-Aldrich, St. Louis, MO, USA) diluted 1:10,000 for 1 h at 37 °C. After washing three times with washing buffer, color reaction was developed with 3,3′,5,5′-tetramethylbenzidine (TMB) substrate (Sigma-Aldrich, St. Louis, MO, USA). The reaction was stopped with 2 M H_2_SO_4_ and plates were read at OD_450 nm_ using an xMark microplate absorbance spectrophotometer (Bio-Rad Laboratories, Philadelphia, PA, USA). Antibody levels in immunized cattle were expressed as OD_450 nm_ values and compared between vaccinated and control cattle using an ANOVA test (*p* < 0.05).

### Data collection and evaluation

Adult engorged female ticks from vaccinated and control animals were daily collected, counted, weighed and assessed for egg laying capacity and fertility to determine the efficacy of the vaccine candidates, employing the following formulae [2, 11].

Effectonthenumberofadultfemaleticks(%DT)=100[1-(NTV/NTC)],

where NTV is the number of adult female ticks in the vaccinated group and NTC is the number of adult female ticks in the control group.

Effectontickweight(%DW)=100[1-(WTV/WTC)],

where WTV is the average adult female tick weight in the vaccinated group, and WTC is the average adult female tick weight in the control group.

Effectontheegglayingcapacity(%DO)=100[1-(PATV/PATC)],

where PATV is the average weight of the eggs per survived tick in the vaccinated group, and PATC is the average weight of the eggs per survived tick in the control group.

Effectonfertility(%DF)=100[1-(PPLOV/PPLOC)],

where PPLOV is the average weight of the larvae per gram of eggs in the vaccinated group, and PPLOC is the average weight of the larvae per gram of eggs in the control group.

Vaccineefficacy(%E)=100[1-(CRT×CRO×CRF)],

where CRT = NTV/NTC, CRO = PATV/PATC and CRF = PPLOV/PPLOC represent the reduction in the number of adult female ticks, egg laying capacity of the survived ticks, and fertility as compared to the control group, respectively.

A Student’s *t*-test with unequal variance (*pi* = 0.05) was used to compare the results of adult female tick number, tick weight, egg weight and egg fertility between vaccinated and control groups.

### Bioinformatic analysis

Membrane spanning domains were predicted by the TMHMM Server at the Center for Biological Sequence Analysis, Technical University of Denmark, DTU (http://www.cbs.dtu.dk/services/TMHMM/; [25]). The two-dimensional representation was made using TMRPres2D (http://bioinformatics.biol.uoa.gr/TMRPres2D/; [38]). Prediction of linear B-cell epitopes was made using the BepiPred-2.0 server (http://tools.iedb.org/bcell/; [24]). The immunogenicity of the receptors was predicted with VaxiJen 2.0 software (http://www.ddg-pharmfac.net/vaxijen/VaxiJen/vaxijen.html) using the 0.5 antigenicity threshold established by default for parasites [13–15].

## Results

### Antigen selection

**Figure 1:**
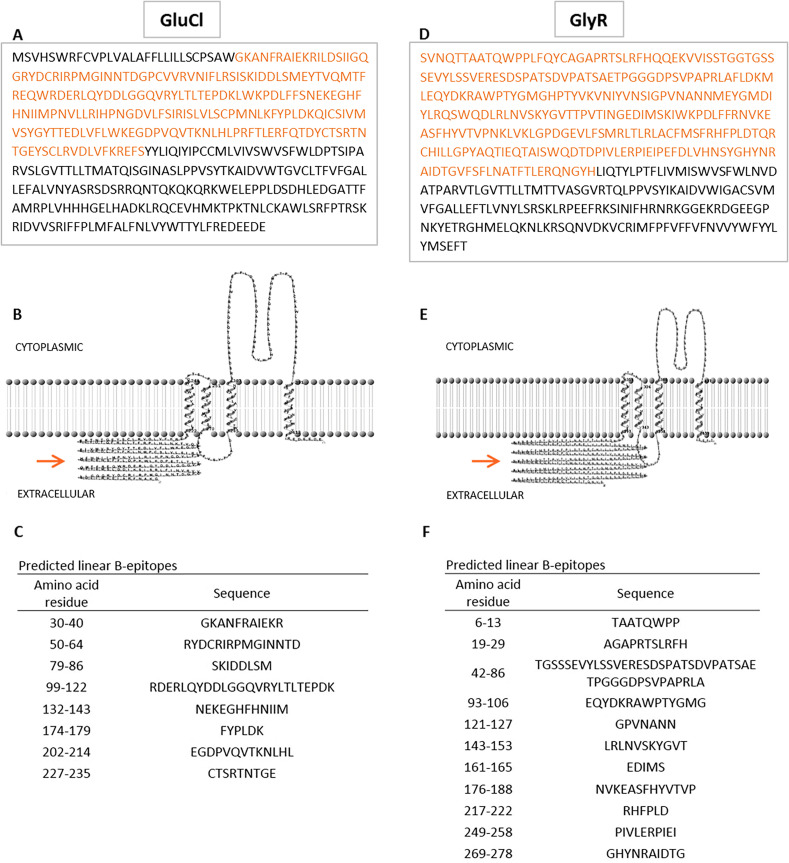
Antigen selection. A. Complete amino acid sequence of a glutamate-gated receptor (GluCl) *from R. microplus* (GenBank accession no. AHE41097.1). N-terminal domain, highlighted by colored letters, was recombinantly produced in *E. coli* and evaluated as a vaccine candidate. B. Two-dimensional representation of GluCl. Arrow indicates the extracellular N-terminal domain. C. Linear B-cell epitopes predicted in N-terminal ECD of GluCl. D. Partial amino acid sequence of a glycine-like receptor (GlyR) from *R. microplus* (GenBank accession no. AHY18971.1). N-terminal domain, highlighted by colored letters, was recombinantly produced in *E. coli* and evaluated as a vaccine candidate. E. Two-dimensional representation of GlyR. Arrow indicates the extracellular N-terminal domain. F. Linear B-cell epitopes predicted in N-terminal ECD of GlyR.

Both GluCl and GlyR, two members of the Cys-loop receptors family of *R. microplus* tick, were selected for the present study, after having fulfilled several requirements. Figure 1 shows the complete amino acid sequence of GluCl receptor (Fig. 1A), the partial amino acid sequence of GlyR receptor (Fig. 1D), and the selected regions to produce recombinant proteins. These regions correspond to N-terminal domains on both receptors and they have an extracellular location (Figs. 1B and 1E). A prediction method of linear B-cell epitopes was applied on these sequences and results showed that the N-terminal ECD sequence of GluCl contained eight possible linear B-cell protective epitopes, whereas the N-terminal ECD sequence of GlyR exhibited eleven potential linear B-cell epitopes (Figs. 1C and 1F). Additionally, both receptors were predicted to be antigenic by using VaxiJen (VaxiJen scores > 0.5).

### Recombinant protein expression

**Figure 2:**
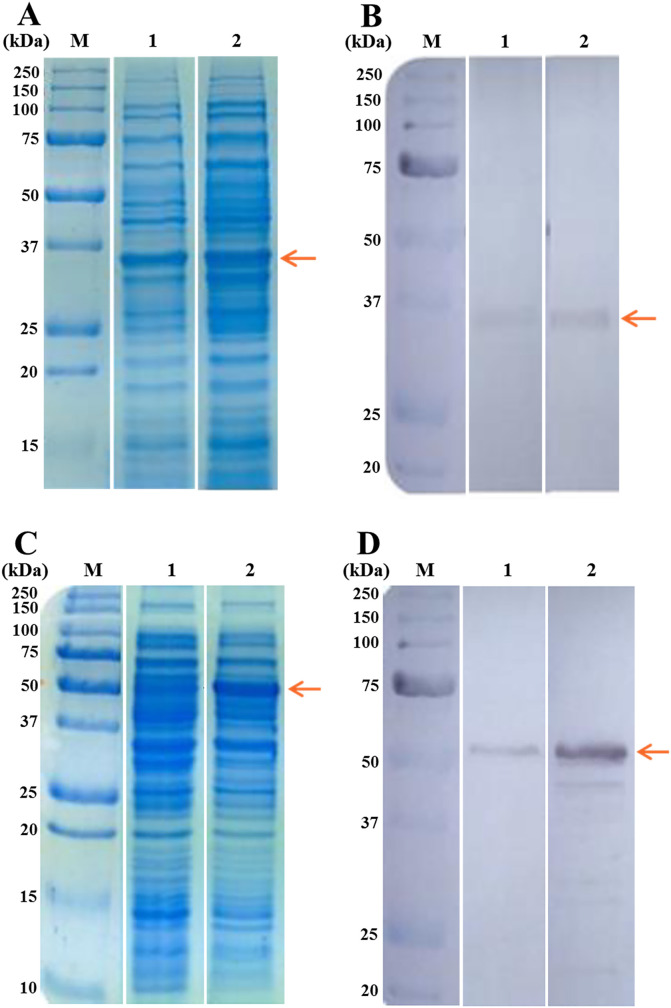
Recombinant proteins expression. A. SDS-PAGE analysis of protein fractions from *E. coli* cultures for rGluCl expression. M = Molecular weight marker. 1 = Uninduced and 2 = Induced culture with IPTG. B. Western blot analysis of rGluCl. M = Molecular weight marker. 1 = Uninduced and 2 = Induced culture with IPTG. C. SDS-PAGE analysis of protein fractions from *E. coli* cultures for rGlyR expression. M = Molecular weight marker. 1 = Uninduced and 2 = Induced culture with IPTG. D. Western blot analysis of rGlyR. M = Molecular weight marker. 1 = Uninduced and 2 = Induced culture with IPTG. In all cases, the molecular weight marker used was the Precision Plus Protein All Blue Prestained Protein Standard from Bio-Rad. Arrows indicate the size of the recombinant proteins.

N-terminal ECD of GluCl and N-terminal ECD of GlyR were recombinantly produced in *E. coli* and purified. Figure 2 shows the SDS-PAGE patterns of protein fractions from *E. coli* cultures during induction of rGluCl and rGlyR expression (Figures 2A and 2C, respectively). Recombinant proteins expression was confirmed by Western blot analyses, which showed a band of ~35 kDa for rGluCl (Fig. 2B), and a band of ~51 kDa for rGlyR (Fig. 2D). Purified recombinant proteins were then used for the immunogenicity studies in mice.

### Mice immunization experiment

**Figure 3:**
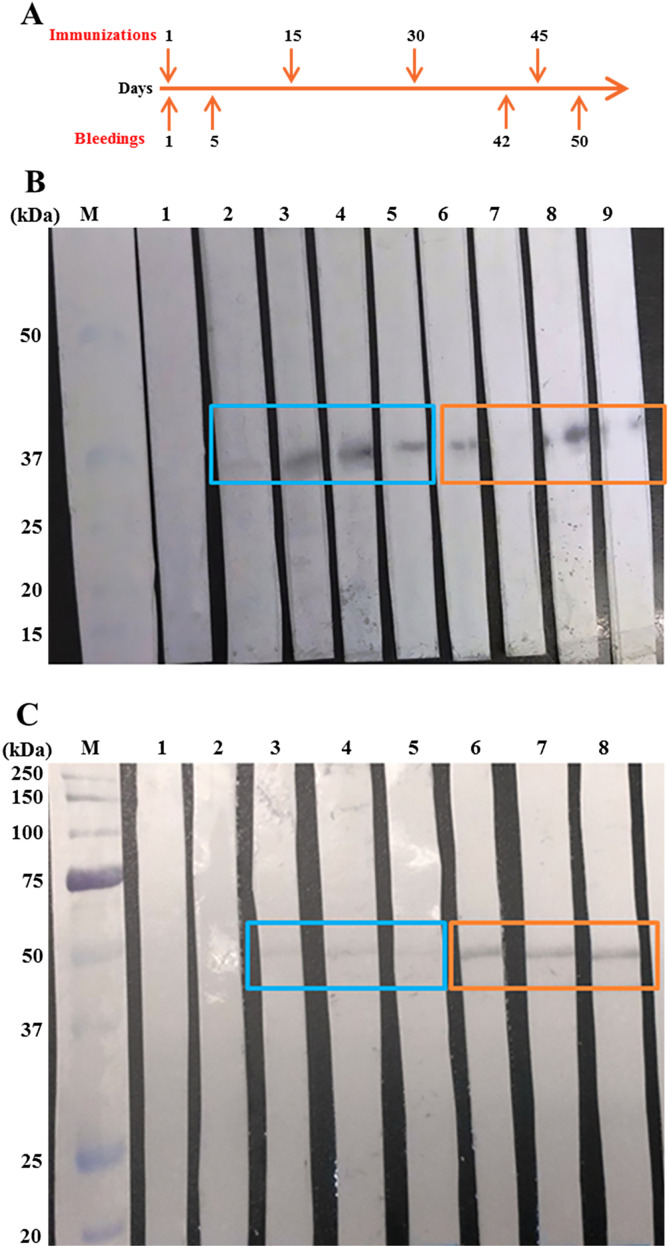
Immunological response in mice vaccinated with the recombinant antigens. A. Immunization and bleeding scheme. B. Immunoreactivity of rGluCl verified by Western blotting. Lane M: protein marker; Lane 1: pooled preimmune sera from 4 mice; Lanes 2–5: individual sera of bleeding 3 from 4 mice; Lanes 6–9: individual sera of bleeding 4 from 4 mice. Immunoreactive bands of ~35 kDa are indicated by rectangles. C. Immunoreactivity of rGlyR verified by Western blotting. Lane M: protein marker; Lane 1: pooled preimmune sera from 3 mice; Lane 2: pooled sera of bleeding 2 from 3 mice; Lanes 3–5: individual sera of bleeding 3 from 3 mice; Lanes 6–8: individual sera of bleeding 4 from 3 mice. Immunoreactive bands of ~51 kDa are indicated by rectangles.

BALB/c mice were independently immunized with the recombinant antigens. Figure 3 shows that both proteins were immunogenic in mice as demonstrated by Western blot assays. Reactive bands were observed only on days 42 and 50 after first immunization. Mice immunized with rGluCl showed a variable immunological response among individuals (Fig. 3B), whereas in mice immunized with rGlyR immunological response was more homogeneous among individuals (Fig. 3C).

### Cattle immunization and infestations experiment

**Figure 4:**
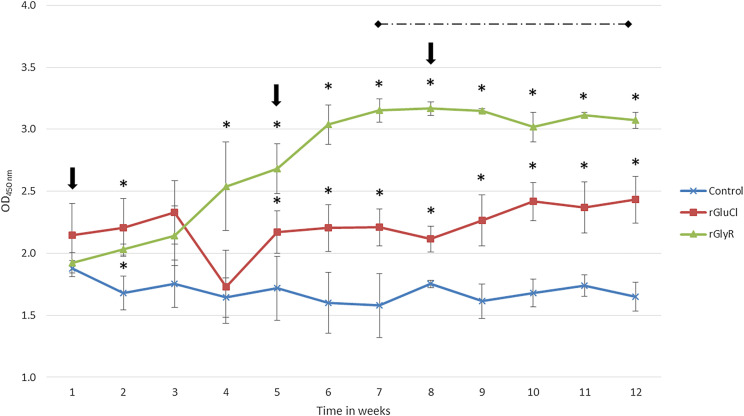
Antibody response in vaccinated cattle. Bovine serum antibody levels to recombinant antigens were determined by ELISA in cattle vaccinated with rGluCl, rGlyR and adjuvant/saline control. Antibody levels in immunized cattle were expressed as OD_450 nm_ values and compared between vaccinated and control cattle using an ANOVA test (*p < 0.05). The time of vaccination shots (arrows) and tick infestations (dashed line) are indicated.

Afterward, recombinant proteins were independently formulated and evaluated in cattle infested with *R. microplus* tick larvae. Vaccination significantly affected the number of engorged adult female ticks on cattle immunized with rGluCl (26.39% reduction) and the group immunized with rGlyR (22.74% reduction), as compared to the control group (Table 1). Reductions in tick weight, egg weight and egg fertility values were also observed in vaccinated groups, although the differences were not significant as compared to the control group (Table 1). Vaccine efficacies of 33% and 25% were calculated for rGluCl and rGlyR, respectively. The IgG immune response elicited in cattle against the recombinant antigens was also determined by ELISA assays (Fig. 4). Results showed that vaccine candidates generated a moderate humoral response on vaccinated cattle, which was more pronounced in cattle vaccinated with rGlyR. Antibody levels increased after successive immunizations and maintained a steady level until the end of the experiment.

**Table 1:** Control of *R. microplus* infestations in cattle vaccinated with the recombinant antigens.

Animal	Total tick number			Tick weight (g)^a^			Egg weight (g)^b^			Egg fertility (%)^c^		
	Control	rGluCl	rGlyR	Control	rGluCl	rGlyR	Control	rGluCl	rGlyR	Control	rGluCl	rGlyR
1	6854	3614	3764	7.09	6.51	6.14	3.70	3.60	3.33	85.81	83.81	83.49
2	5399	3851	4938	6.55	5.73	7.11	3.48	3.02	3.67	84.12	80.16	84.90
3	2957	3733	3050	5.52	5.20	5.54	2.91	2.79	3.01	83.53	83.43	81.78
Mean ± SD	5070 ± 1607	3732 ± 96	3917 ± 778	6.39 ± 0.65	5.81 ± 0.54	6.26 ± 0.65	3.36 ± 0.33	3.14 ± 0.34	3.34 ± 0.27	84.49 ± 0.97	82.47 ± 1.64	83.39 ± 1.28
% reduction	–	26.39*	22.74*	–	9.08	2.03	–	6.55	0.59	–	2.39	1.3
*t*-test^d^	*P < 0.05											
% efficacy^e^	rGluCl – 33%											
	rGlyR – 25%											

a Mean weight of 20 tick specimens.

b Mean egg weight from 20 tick specimens.

c Mean hatching percentage of eggs from 20 tick specimens.

d Total tick number, tick weight, egg weight and egg fertility values were compared by Student’s *t*-test with unequal variance between vaccinated and control groups (*p < 0.05).

e Vaccine efficacy was calculated as 100[1 − (CRT × CRO × CRF)], where CRT, CRO and CRF are the reduction in the number of adult female ticks, oviposition and egg fertility as compared to the control group, respectively.

## Discussion

Considerable efforts have been devoted toward identifying anti-tick vaccine antigens [8, 30, 35]. In this sense, however, transmembrane proteins have not yet been fully exploited in vaccination studies, despite the fact that their vaccine efficacy has been demonstrated through Bm86 orthologs and aquaporin-based vaccines [8].

In this study, we tested the immunogenicity and protective efficacy of recombinant N-terminal ECD of two members of Cys-loop receptors, namely, GluCl and GlyR of *R. microplus* tick, following a reverse vaccinology approach. These candidates were evaluated on the basis that they fulfilled a series of selection criteria related to their expression level, biological function, accessibility and subcellular localization [8, 26, 34]. Moreover, both receptors were predicted to contain several potential linear B-cell epitopes and to be antigenic. VaxiJen software is an immunoinformatic tool that has been widely used to identify subunit vaccine candidates among proteins of bacterial, viral, parasite, fungal and tumor origin [43]. Recently, VaxiJen was used to identify putative protective antigens in *R. microplus* ticks. Maritz-Olivier et al. [26] identified 791 vaccine candidates, of which 176 were membrane-associated and 86 secreted soluble proteins. The authors performed a preliminary analysis on the antigenicity of 5 membrane-associated proteins using polyclonal antisera from BALB/c mice immunized with a crude extract of tick midgut membrane proteins, and found that candidates had an IgG binding capacity greater than previously identified epitopes of Bm86, although vaccination trials were not carried out. On the other hand, Richards et al. [35] identified 21 transmembrane proteins as having a VaxiJen score ≥ 0.5. Interestingly, a putative calnexin showed a higher VaxiJen score (0.91) than that predicted for Bm86 (Vaxijen score: 0.77); however, cattle vaccine trials that validate their findings are still lacking.

Once selected, our vaccine candidates were recombinantly produced in *E. coli* and purified. Recombinant proteins were successfully expressed and obtained at the expected sizes (Fig. 2). It is worth mentioning that intense bands were observed on IPTG-induced cultures. However, faint bands were also observed on un-induced cultures. A possible explanation could be that the pET32 plasmids we used contain the T7 promoter, which is known for having background (“leaky”) expression [28, 39, 42].

With regard to immunization experiments, our results showed that rGluCl and rGlyR were immunogenic both in mice and cattle, as evidenced by Western blot and ELISA assays, respectively (Figs. 3 and 4). In cattle, a moderate humoral response against vaccine candidates was observed (Fig. 4). A decreased anti-rGluCl antibody response was detected in week 4, which was recovered after booster immunizations. The cause for this decrease is unknown but it could be related to problems associated with the stability of the vaccine formulation, as suggested by Almazán et al. [2]. From other studies, it is known that a direct correlation exists between antibody titers to certain tick antigens and the efficacy of vaccination [10]. Nevertheless, this is not necessarily true for other tick antigens. Several studies have reported potent antigen-specific antibody responses that did not correlate with the expected protection levels [3, 4, 23]. In our study, antibody levels are consistent with the obtained vaccine efficacies. However, antibody levels were higher against rGlyR than rGluCl, although higher anti-*R. microplus* efficacy was obtained against the latter. rGlyR was apparently more immunogenic than rGluCl but afforded less protection, confirming previous observations that antibody titers do not always match with efficacy. The modest immunogenicity may be a consequence of the bacterial recombinant protein expression system used. Tellam et al. [40] have demonstrated that vaccine formulations containing the insect cells-expressed Bm86 have higher efficacy for the control of tick infestations than those made from *E. coli*-expressed antigens. Furthermore, glycosylated recombinant Bm86 purified from *Pichia pastoris* was demonstrated to be more immunogenic that non-glycosylated one [12]. This suggests that glycosylation on tick proteins may be important for enhancing both immunogenicity and protective efficacy of tick antigen formulations [12]. It is worth mentioning that both receptors in this study have multiple potential glycosylation sites (data not shown). Therefore, their production in a eukaryotic system could increase their immunological properties.

On the other hand, vaccination in cattle significantly affected the number of engorged adult female ticks, whereas it had no significant effects on tick weight, egg weight and egg fertility (Table 1). Obtained vaccine efficacies are similar to those of several tick antigens previously tested against *R. microplus* ticks, such as Bm86-CG, trypsin inhibitor, ATAQ peptide, EF1a, Bm91, VTDCE and BYC [1, 5, 8, 9, 30]. Several authors have proposed that combining antigens (cocktail anti-tick vaccines) could improve efficacies of individual vaccines. However, this still requires experimental validation [30]. We recognize that our findings are limited by the small number of animals used in the controlled pen trial. Because bovine individual response to both tick infestation and vaccination is heterogeneous (Table 1), additional trials with more animals are required to reach a conclusion about the usefulness of tested antigens for the control of cattle tick infestations.

Finally, the followed reverse vaccinology approach offers several advantages over the traditional method, such as the speed in the identification of putative protective antigens and this type of approach could obviate the need to use non-bovine animal models to determine if a recombinant tick antigen is immunoprotective, holding the promise of being able to predict novel potential tick vaccine candidates. However, to improve such predictions, several considerations must be taken into account. Diverse epitope prediction programs use human or murine major histocompatibility alleles, excluding bovine data sets, which may yield misleading information [7, 26, 35]. With regard to the VaxiJen program, it has been until very recently applied to ectoparasites, generating an large number of putative tick protective antigens [26, 35]. Vaccination trials will discriminate protective from non-protective antigens, and this s should improve the predictive potential of VaxiJen for the identification of additional anti-tick vaccine candidates.

## Conclusions

To our knowledge, this study reports for the first time the targeting of nervous system components of *R. microplus* cattle ticks through vaccination. Recombinant N-terminal Cys-loop receptors domains were immunogenic and conferred partial protection against *R. microplus* tick infestations. Changing the recombinant protein expression system to a eukaryotic one could improve the efficacy of vaccine candidates.

## Conflict of interest

The authors declare that they have no conflict of interest.
